# Eye, Ear, Nose, and Throat

**Published:** 1895-05

**Authors:** 


### EYE, EAR, NOSE, AND THROAT.

EDITED BY DUNBAR ROY, A.B., M.D.

### THE USE OF CONVEX GLASSES FOR DISTANT VISION IN MYOPIA.

We propose to discuss the advantage to be gained in certain cases of myopia from the use of convex glasses for distant vision. Although this seems at first paradoxical, it is unquestionably true that myopes succeed in considerably increasing their sharpness of vision and are enabled to read characters at a distance which they could not distinguish with concave glasses, such, for example, as the name of a street, or the number of a house, by placing at some distance in front of the better eye a convex glass, as they would use a lorgnette. The inconvenience of this procedure is in the fact that an inverted image is obtained; but with a little practice inverted characters are easily read, and the facts prove conclusively that this inconvenience is amply compensated for by the increased size of the image, since we know of myopic persons who make habitual use of such glasses.

The method of employing convex glasses in myopia consists in placing the glass before the eye at such a distance that its focus coincides with the *punctum remotum.* If the myope completely relaxes his accommodation, as he must do under other circumstances, he finds that with the aid of this convex glass the eye is adjusted for parallel rays and can see at a distance. An inverted image is thus obtained, just as the ophthalmoscopic observer receives an inverted image of a myopic eye when, withdrawing from the eye under examination, he adjusts for the *punctum remotum* of the observed eye, either by the aid of his own accommodation or by the use of a convex lens.

The amount of enlargement of the images of objects observed depends on the amount of myopia and the focus of the convex glass employed. With a given convex lens, the image is enlarged in proportion as the degree of myopia is higher. On the other hand, in equal degrees of myopia the amplification of the image increases with the use of weaker convex lenses. The use of weak convex lenses, in high degrees of myopia, to obtain considerable enlargement, is thus rendered impracticable by the fact that it is necessary to hold the glass at quite a distance from the eye, and the image is so enlarged that the field of vision becomes contracted and a slight movement of the hand which holds the glass results in the disappearance of the object observed.

Practically, glasses weaker than five dioptries cannot be employed, and it is desirable that they should be five or six centimeters in diameter. Under these conditions, the myope, with a little practice, can easily see the objects which he desires to observe, and the visual acuity will be found to be more than double that which is obtained by correcting the myopia with a convex glass, as we have recently had occasion to observe in a case of a person with myopia of twenty dioptries who had accustomed himself to the use of a convex lens.

It will be noticed that with convex lenses a phenomenon occurs exactly opposite to that which is observed in a myopic eye supplied with a concave lens, as in the latter case objects appear diminished in size (a fact which persons having both a myopic eye and an emmetropic or a hypermetropic eye can easily prove); on the other hand, if the myopia is corrected with a convex lens there is an enlargement of the image, and if the convex lens employed is suitable, the advantages are far superior to those obtained with spectacles.

In conclusion, we think it would be advantageous to extend the use of the convex glasses to many cases where, with a high degree of myopia, the visual acuity is defective. Glasses of about + 6 dioptries should be prescribed and used like the magnifying glass which certain people use for reading; they thus constitute a magnifying glass for distant vision. Since these glasses produce an inverted image, they are to be used only occasionally, when the myope wishes to see with precision small objects, such, for example, as a public notice, the name of a street, or the number of a house. In these special instances convex lenses will be regarded by certain myopic persons as quite superior to concave lenses, and they will be thankfully accepted as a valuable expedient.

Having had the opportunity of discussing this optical question with Dr. G. Poullain, our colleague desires to add the following note to our communication :

"I have made similar experiments," says Dr. Poullain, "and have been able to increase the visual actuity in several cases ot myopia of a high degree; I have also made some attempts at obtaining an erect image.

"I at first attached a convex lens to one of the faces of the right angle of a rectangular prism, and by looking through the other face of the prism, held at a fixed distance from the eye, I was able, on making myself myopic, to see objects erect, but with this inconvenience, that the right side of the object was seen at the left, and *vice versa,* for the total reflection on the surface of the hypothenuse only caused the image inverted by the lens to be erect in the plane perpendicular to the faces of the prism.

"I then made the experiment of fastening two prisms together by one of the right-angle faces in such a way that the edge of one prism was perpendicular to the edge of the other. In this way the image of the object underwent the double erection required. Instead of attaching a convex lens to the incident surface only, I attached one also to the surface of emergence, the refraction of the two lenses being equal to that of the single lens of the first experiment; in this way either face could be used as the incident surface. But with this apparatus, only objects situated at the right or left could be seen, and considerable experience was necessary in order to make use of it. I preferred an arrangement similar to that of Porro's telescope.

"Half of the hypothenuse face of a right-angled prism was attached to half of the same face of another right-angled prism. On each of the free portions of the hypothenuse faces was attached a lens of two and a half dioptries, and by placing this biprism at a proper distance—that is, such that its focus was at the far point of the myopic eye—objects appeared *erect* and *enlarged.*

"Other things being equal, the field increases as the square of the face of emergence, and the weight of the biprism varies as the cube of the same side. This consideration raises an obstacle to the use of such a biprism: if it is too small the field will be too limited, and if it is large it will be too heavy.

"I have lately found the experiment on which this communication is based in the Optics of Kepler, Proposition LXXVIII.

This scientist, who had a myopia of high degree, without doubt found in his own case this peculiarity of a myopic eye, and I am inclined to think that it was the origin of the invention of his astronomical telescope. He understood that to place a normal eye in the condition of his own, it sufficed to add to that eye the refractive power which he had in excess.

"In conclusion, I desire to call attention to the phenomena which occur in a hypermetropic eye under similar conditions—that is, in looking through a lens a number of dioptries less than the measure of his own ametropia and placed at a proper distance from the eye. Under such circumstances, the hypermetrope sees objects *enlarged* and *erect*, thus simulating Galileo's telescope, as in Kepler's experiment the excess of refraction of the myopic eye replaced the convex ocular of the astronomical telescope."—*De Wecker and Masselon, Ann. d'Oculistique, February,* 1895.

---

### ALBUMINURIC RETINITIS OF PREGNANCY.

All the peculiarities of albuminuric retinitis in pregnant women are not yet well known. Dr. Silex is in possession of preparations which show the vessels intact.

Its symptomatology and prognosis are very inexactly treated in literature, and its therapeutics have been much neglected. The author has given much attention to this special pathological subject for many years, and has arrived at definite conclusions in regard to the affection.

To form a judgment of a case, it is necessary first to make a critical ophthalmoscopic examination of the retinal arteries. In the first stages of the disease, the central artery is often seen with the erect image transformed into a large white cord. This is produced by dilatation of the perivascular lymphatic space. This change may disappear, but hyaline degeneration of the arteries threatens the canals of the vessels, which may compromise the function of the internal layers of the retina and produce atrophy of this membrane and of the optic nerve. If the vessels are normal, albuminuric retinitis presents little danger. This form of retinitis may arise from affection of the kidneys during pregnancy, an acute or chronic nephritis, the general symptoms of which are early developed. The visual disturbances develop slowly, most frequently in the second period of pregnancy, especially in primiparæ. If they develop rapidly, chronic nephritis must be assumed. After delivery, vision is established more or less completely.

In twenty-two patients examined during a considerable period, eleven recovered vision more than one-sixth, ten others remained with a lesser degree of acuity, and five of them were almost blind. This unfortunate result was due to atrophy of the optic nerve, to retino-choroiditis, and to detachment of the retina, which occurred at a later stage in both eyes. These figures are so important from a social point of view, that Silex advises interference with pregnancy in all cases where retinitis is found. Its prognosis is indeed uncertain; the women are frequently attacked with eclampsia, and the hope of a living child is but faint. In chronic nephritis, danger to the life of the mother and child settles the question of interference; in case of nephritis of pregnancy, premature delivery is not advocated, as in several cases good vision has returned after expectant treatment.

The condition of the retinal vessels should decide the question of interference. For example, in two pregnant women, at the commencement of the eigth month, one having an acuity of six-eighteenths, and the other of one-eighteenth, one might be authorized to perform premature delivery in the first case on account of the vascular changes, while one should wait in the second. It is interesting that Dr. Silex has been able to find large quantities of albumin in the urine during one or two years without development of chronic nephritis in several women in whom the retinal lesion had disappeared and the general health had remained good. The long duration of albuminuria has not always a bad prognosis when it can be attributed to a renal affection of pregnancy.—*Ann. d'Oculistique.*

